# Comparative temporal metabolomics studies to investigate interspecies variation in three *Ocimum* species

**DOI:** 10.1038/s41598-020-61957-5

**Published:** 2020-03-23

**Authors:** Shubhra Rastogi, Saumya Shah, Ritesh Kumar, Ajay Kumar, Ajit Kumar Shasany

**Affiliations:** 1 Centre for Biotechnology, Shiksha ‘O’ Anusandhan University, Bhubaneswar, 751003 Odisha India; 2 Biotechnology Division, CSIR- Central Institute of Medicinal and Aromatic Plants, PO CIMAP, Lucknow, 226015 UP India

**Keywords:** Plant molecular biology, Secondary metabolism

## Abstract

*Ocimum* is one of the most revered medicinally useful plants which have various species. Each of the species is distinct in terms of metabolite composition as well as the medicinal property. Some basil types are used more often as an aromatic and flavoring ingredient. It would be informative to know relatedness among the species which though belong to the same genera while exclusively different in terms of metabolic composition and the operating pathways. In the present investigation the similar effort has been made in order to differentiate three commonly occurring *Ocimum* species having the high medicinal value, these are *Ocimum sanctum*, *O. gratissimum* and *O. kilimandscharicum*. The parameters for the comparative analysis of these three *Ocimum* species comprised of temporal changes in number leaf trichomes, essential oil composition, phenylpropanoid pathway genes expression and the activity of important enzymes. *O. gratissimum* was found to be richest in phenylpropanoid accumulation as well as their gene expression when compared to *O. sanctum* while *O. kilimandscharicum* was found to be accumulating terpenoid. In order to get an overview of this qualitative and quantitative regulation of terpenes and phenylpropenes, the expression pattern of some important transcription factors involved in secondary metabolism were also studied.

## Introduction

*Ocimum* is one of the highly important medicinal plants used worldwide in the traditional medicinal systems. It is commonly known as ‘basil’ but is popular as ‘Tulsi’ in India. Not only in medicine, basil also has religious importance^1,2^. *Ocimum* belongs to family Lamiaceae and subfamily Nepetoideae which contains strongly fragrant plants marked by the presence of essential oils comprising of sesquiterpenes, monoterpenes and phenylpropanoids. Eugenol, methylchavicol and linalool are the major compounds found in most basil species, however, the composition of every chemical constituent differ in different species or varieties^1^. Another feature in the plants of Lamiaceae family is the presence of oil glands called glandular trichomes on the leaf surface. Mainly there are two types of oil glands^3^ that is, peltate and capitate. The glands are differentiated by the number and size of head cells occurring on the basil leaf surface, and the phenylpropenes are exclusively synthesized in the peltate glands^4^. Surplus glandular trichomes in plants and their association with essential oil biosynthesis in numerous plants including peppermint^5–14^, lima bean^15–18^, and tomato^19–21^ have been talked about, which has improved our comprehension of isoprenoid production in plants. The general phenylpropanoid pathway produces a wide range of secondary metabolites originating from the shikimate pathway intermediates. However, the role and impact of this gene family need to be established^22^. Phenylpropanoids contribute to both abiotic and biotic plant stress responses. Apart being the indicators of stress, they also help in acquiring resistance against pests^23^. They support the incursion of fresh habitation^24^ and help in providing the biochemical sources for successful reproduction^25^. Phenylpropenes form a collection of small phenolic molecules which result from the general phenylpropanoid biosynthetic pathway, and are the main flavoring factors in a lot of spices and herbs, like- nutmeg, cloves, cinnamon, pimento, allspice, tarragon and basil. Besides this, phenylpropenes (like eugenol, methyleugenol etc.) are the key constituents of the protective mechanism in plants and even help in attracting the pollinators for pollination^9^. One of the most important reservoirs of phenylpropenes is the ‘Holy basil’ whose whole genome has recently been sequenced. Thus, an effort to study the temporal expression of phenylpropanoid pathway genes in *O. sanctum* (OS), the study was designed so as to compare the expression pattern of another phenylpropene rich species, *O. gratissimum* (OG) and a terpene producing species, *O. kilimandscharicum* (OK). The differences in oil composition, trichome number and also the expression of the genes governing the phenylpropanoid biosynthesis as well as the expression pattern of transcription factors involved in secondary metabolism were studied in these three *Ocimum* species. Since the synthesis of secondary metabolites as well as the transcripts of respective biosynthesis pathway genes is tightly regulated at various levels^26^, therefore, transcription factors governing the process of transcription have also been investigated. Characteristics of the three species are discussed here. *O. sanctum* (*tenuiflorum*), is a grand sacred medicinal plant of India, commonly known as holy basil or Tulsi. Basically there are two types of varieties (i) Green leaves tulsi plant- Sri/Rama Tulsi and (ii) Purple leaves tulsi plant- Krishna/ Shyama Tulsi^27^. Traditional medical practitioners widely use *O. sanctum* to treat various ailments like bronchial asthma, bronchitis, dysentery, malaria, arthritis, diarrhea, chronic fever, skin diseases, insect bite, etc. In addition, it acquires antifertility, antidiabetic, anticancer, antimicrobial, antifungal, cardioprotective, hepatoprotective, antiemetic, analgesic, antispasmodic, diaphoretic and adaptogenic activities^28–30^. Holy basil possesses a highly-flavored and a bit pungent savor in comparison to other basils because of a phenylpropanoid, eugenol and a sesquiterpenoid, *beta* caryophyllene present in the leaf essential oil. The application of essential oil has demonstrated the reduction in inflammation, joint pains as well as body rashes^29^. *O. gratissimum* (wild basil, tree basil or clove basil) is a perennial herb, aromatic in nature and found to occur in tropical regions, like- India and West Africa^31^. It is popular in India as *Vana tulsi* (Hindi) or *Vriddhutulsi* (Sanskrit)^32^. Though pungent in taste, it is helpful in treating brain, liver, heart and spleen related ailments, as well as strengthen the gums and eliminates foul breath smell^33^. It has also been demonstrated to acquire diaphoretic, laxative, antidiarrhoeal, antipyretic, anti-inflammatory, immunostimulatory, cardiovascular, hepatoprotective, antidiabetic, wound healing, antihypertensive, analgesic and anticonvulsant effects^29,34^. *O. gratissimum* contain essential oil with a high percentage of eugenol. A variation is found in the chemical composition of the oil and 6 chemotypes based on oil composition variation have been reported. Chief components present in the *O. gratissimum* essential oils are- eugenol, citral, thymol, geraniol, ethyl cinnamate and linalool^35^. Essential oil finds application in food, flavor, beverages, dental preparations and detergents *etc*^29^*. O. kilimandscharicum* Guerke, known as ‘Kapoori Tulsi’ (Hindi) or ‘Camphor Basil’ (English) is a foreign west African species native of East Africa which was brought to India and Turkey. This plant attracted attention as a source of camphor^36^. This species has a strong but less pleasant flavor. The plant has carminative, stimulant, antipyretic, anti-fungal and anti-bacterial properties. Conventionally, in East Africa, plant extracts of *O. kilimandscharicum* were employed to treat many diseases like cold, cough, diarrhea, measles, abdominal pains as well as it had inhibitory action against mosquitoes and pest^37^. The leaves of the plant are aromatic, antiviral, thermogenic, acrid, anti-bacterial, appetizing, insecticidal, and ophthalmic^36^. The color of essential oil is light yellow and owes a strong camphory odour, used widely in perfumery, flavor and pharmaceutical industries.

Temporal variation in the essential oil yield has been attributed to the environmental factors; however, till now there has been no investigation conducted to observe the status of essential oil pathway gene expression and/ or the expression of the regulatory elements, like- the transcription factors. Additionally, the differences among the species of *Ocimum* have only been established on the basis of essential oil composition and constituents. Therefore, this study was planned to give a comparative overview of the three *Ocimum* species based on the effect on essential oil yield with time as well as the differences in biochemical constituents like- chlorophyll, phenolics, anthocyanin and activity of some phenylpropanoid pathway enzymes *viz*. PAL (phenylalanine ammonia lyase), C4H (cinnamic acid 4-hydroxylase), 4CL (4-coumarate CoA ligase) and CAD (cinnamyl alcohol dehydrogenase). Simultaneously, the essential oil profiling and trichome density measurement was also carried out every month to see the effect of time over them.

## Results

### Chemoprofiling of essential oil

**Figure 1:**
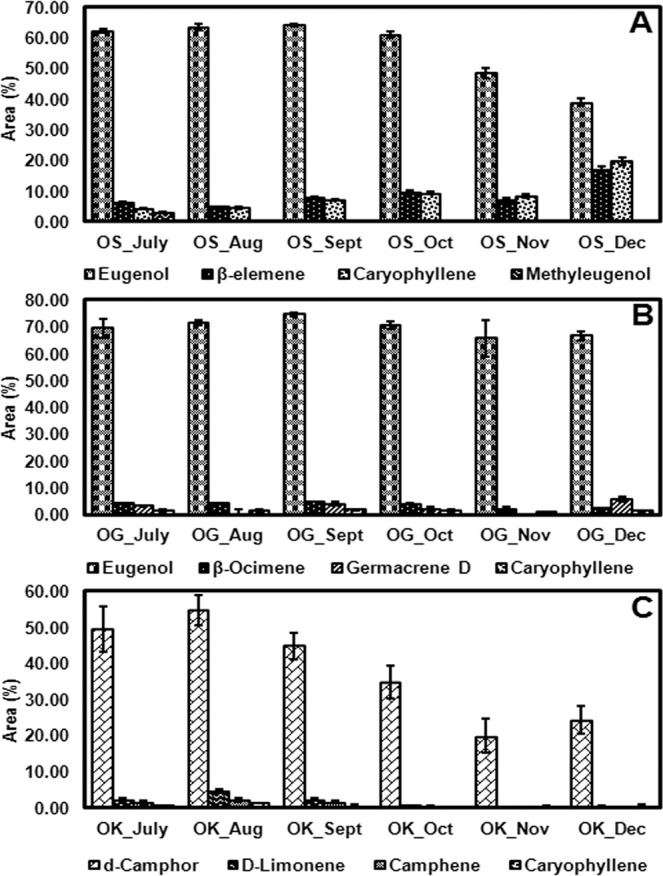
Abundant essential oil constituents from six months oil profiling of (**A**) *Ocimum sanctum* (OS) (**B**) *Ocimum gratissimum* (OG) and, (**C**) *Ocimum kilimandscharicum* (OK). Data are given as means ± SD (at least three independent replicates).

Aiming at the investigation of the seasonal variation, the leaves of *O. sanctum, O. gratissimum* and *O. kilimandscharicum* were collected in triplicates throughout one year in the months of July, August, September, October, November, and December 2018. The yields of the leaf essential oils varied between 0.29 and 0.87% (w/ w) for *O. sanctum*, 0.11 to 1.3% (w/w) for *O. gratissimum* and between 0.1 and 0.73% (w/w) for *O. kilimandscharicum* (Table 1). Total compounds which are represented in metabolic profile were those having peak area percent greater than and equal to 0.01 were considered for metabolic profiling analysis (39 compounds from *O. sanctum*, 47 from *O. gratissimum* and 22 from *O. kilimandscharicum*). As evident from Fig. 1, abundant essential oil constituents (d-camphor, D-limonene, camphene, and caryophyllene) in *O. kilimandscharicum* were found to be present in higher amounts from July to October and thereafter the contents of these constituents started decreasing. Similar pattern of essential oil constituents (eugenol, β-elemene, caryophyllene and methyleugenol) was also observed in the oil profiling from the leaves of *O. sanctum*. However, no major effect of time and environmental factors was observed in *O. gratissimum* essential oil constituent profiling (eugenol, β-ocimene, germacrene-D and caryophyllene) (Fig. 1). Eugenol was identified to be present in the highest concentration in the leaf essential oils of both *O. sanctum* and *O. gratissimum* while camphor constituted the major proportion of the *O. kilimandscharicum* oil. As per the oil profiling (Supplementary Table 3) of the *Ocimum* varieties taken in the present study *O. sanctum* and *O. gratissimum* were found to be rich in phenylpropenes while *O. kilimandscharicum* was rich in terpenes. The essential oil constituents were categorized under five major class of compounds namely, phenylpropenes, monoterpenes, monoterpene alcohols, sesquiterpenes and sesquiterpenoid alcohols. The array of essential oil constituents was highest in *O. gratissimum* followed by *O. sanctum* and was lowest in *O. kilimandscharicum* (Supplementary Table 3).

**Table 1:** Essential oil yield of the three *Ocimum* species for six months.

Months	*O. sanctum* (%)	*O. gratissimum* (%)	*O. kilimandscharicum* (%)
July	0.41 ± 0.11	0.64 ± 0.08	0.48 ± 0.14
August	0.35 ± 0.05	0.83 ± 0.08	0.73 ± 0.17
September	0.68 ± 0.11	1.12 ± 0.11	0.59 ± 0.06
October	0.87 ± 0.03	1.30 ± 0.40	0.42 ± 0.07
November	0.47 ± 0.04	0.35 ± 0.10	0.26 ± 0.07
December	0.29 ± 0.07	0.11 ± 0.01	0.10 ± 0.02

Data are means ± SD (at least three replicates).

### Glandular trichome density

**Figure 2:**
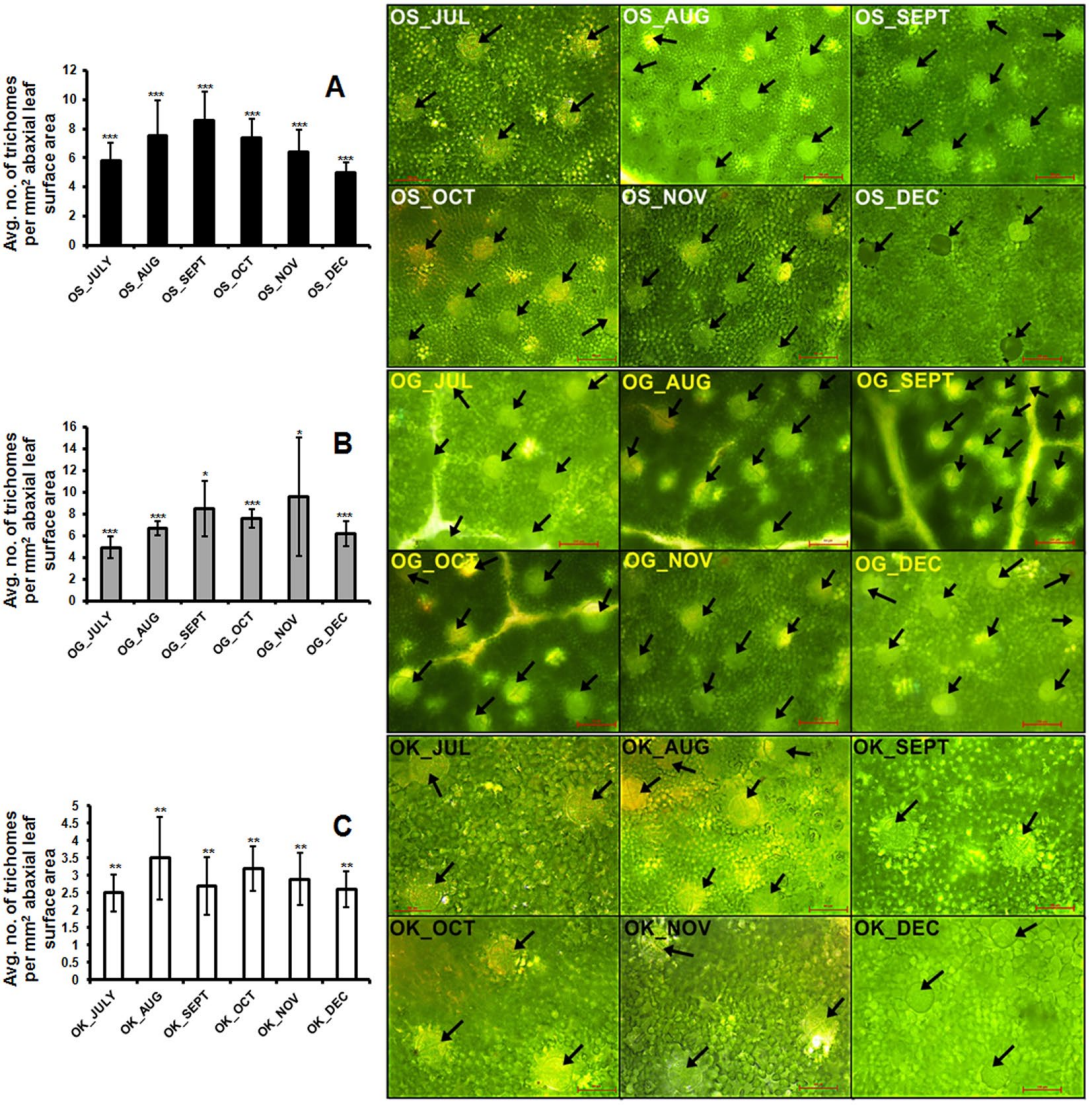
Trichome densities and their representative microscopic images for six months in three panels for (**A**) *Ocimum sanctum* (OS) (**B**) O*cimum gratissimum* (OG) and, (**C**) *Ocimum kilimandscharicum* (OK). Statistical analysis was performed using HSD tukey test, standard weighted-means analysis at *P < 0.01, **P < 0.05 and ***P < 0.1 levels of significance. Arrows in the figure are pointing the glandular trichomes on the abaxial leaf surface; Scale bar = 100 µm. Error bars represent ± SD (at least ten independent plants).

Trichome density in terms of average number of trichomes per mm^2^ leaf area were recorded from the abaxial surface of leaves in all the six months from July to December in a population size of 10 plants each month for *O. sanctum*, *O. gratissimum* as well as *O. kilimandscharicum* (Fig. 2). Highest effect of variation was found to be observed in *O. sanctum* species where the trichome density gradually increased from July to September and decreased thereafter onwards October. In *O. gratissimum* also the trichome density increased from July to September but fluctuated in October and November and finally got decreased in the month of December. However *O. kilimandscharicum* was found to be having least temporal variation on its trichome densities. Trichome densities were observed to be highest during the months of September, November, and August in *O. sanctum, O. gratissimum* and *O. kilimandscharicum*, respectively. *O. gratissimum* and *O. sanctum* were found to be having more number of peltate glandular trichomes in comparison to *O. kilimandscharicum*.

### Phenylpropanoid pathway gene expression studies

**Figure 3:**
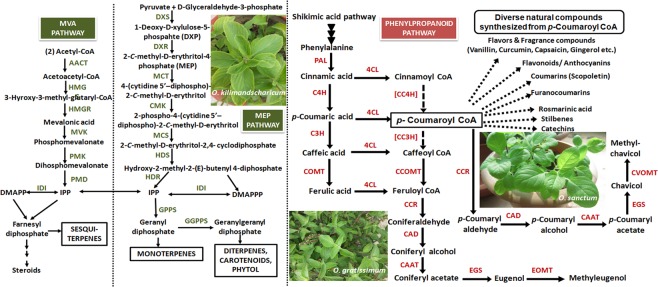
Pathways leading to the generation of terpenes and phenylpropenes in *Ocimum* sp. giving an overview of the MVA (mevalonate) and MEP (2-C-methyl-D-erythritol-4-phosphate) pathways localized in the cytosol and the plastids, respectively. DXS, DXP synthase; DXR, DXP reductoisomerase; MCT, 2-C-methyl-D-erythritol-4-(cytidyl-5-diphosphate) transferase; CMK, CDP-ME kinase; MCS, CMEPP synthase; HDS, HMBPP synthase; HDR, hydroxy-2-methyl-2-(E)-butenyl 4-diphosphate reductase; IDI, IPP isomerase; AACT (acetoacetyl-CoA thiolase), HMGS (HMG synthase), HMGR (HMG reductase), MVK, MVA kinase; PMK, phosphome-valonate kinase; PMD, MVA diphosphate decarboxylase; and GGPPS, GGPP synthase; C4H, cinnamate 4-hydroxylase; 4CL, 4-coumarate: CoA ligase; C3H, *p*-coumarate 3-hydroxylase; COMT, caffeoyl O-methyl transferase; CC4H, cinnamoyl-CoA 4-hydroxylase; CC3H, p-coumaroyl- CoA 3-hydroxylase; CCOMT, caffeoyl-CoA O-methyl transferase; CCR, cinnamoyl-CoA reductase; CAD, cinnamyl alcohol dehydrogenase; CAAT, coniferyl alcohol acetyl transferase; EGS, eugenol synthase; EOMT, eugenol O-methyl transferase and CVOMT, chavicol O-methyl transferase. The enzymes for 4- and 3-hydroxylation of CoA esters have not been demonstrated yet and hence [CC4H] and [CC3H] are indicated in brackets to indicate their probable role (Gang *et al*.^56^) [adapted from Iijima *et al*.^41^ and Rastogi *et al*.^38^].

**Figure 4:**
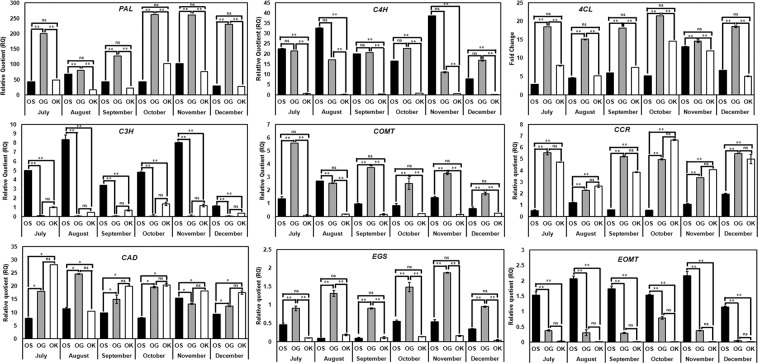
Relative expression levels of PAL, C4H, 4CL, C3H, COMT, CCR, CAD, EGS and EOMT transcripts in *O. sanctum* (OS)*, O. gratissimum* (OG) and *O. kilimandscharicum* (OK) during six months starting from July’ 2018 till December’ 2018 determined by Real-time RT-PCR. Data are means ± SD (for at least three replicates), and the y-axis represents the relative quotient (RQ). Statistical analysis was performed using HSD tukey test, standard weighted-means analysis at P < 0.01* and P < 0.05** levels of significance while ‘ns’ means non-significant.

Quantitative real-time PCR (qRT–PCR) analysis was performed to assess the transcript levels of nine important phenylpropanoid pathway genes in glandular trichomes at different developmental stages collected throughout one year in the months of July, August, September, October, November, and December 2018. The nine genes taken in the present study are- phenylalanine ammonia lyase (*PAL*), cinnamic acid 4-hydroxylase (*C4H*), 4- coumarate CoA ligase (*4CL*), *p*-coumarate 3-hydroxylase (*C3H*), caffeic acid *O-* methyltransferase (*COMT*), cinnamoyl CoA reductase (*CCR*), cinnamyl alcohol dehydrogenase (*CAD*), eugenol synthase (*EGS*), and the eugenol *O-* methyltransferase (*EOMT*). Analysis was performed by designing the primers from the genes reported in the NCBI database as well as from transcriptome and the whole genome sequencing data of *O. sanctum* reported recently^38–40^ (Supplementary Table 1). Figure 3 shows the detailed pathway leading to biosynthesis of terpenes and phenylpropenes in the glandular trichomes of the three *Ocimum* species as earlier discussed by Iijima *et al*.^41^ The results reveal that overall there is a higher expression of phenylpropanoid pathway genes in *O. gratissimum* as compared to *O. sanctum* and *O. kilimandscharicum*. In the succession of gene expression, the highest expression in terms of relative quotient (RQ) was observed in *PAL* followed by *CAD, C4H, 4CL, C3H, CCR, COMT, EOMT* and *EGS* (Fig. 4).

Expression of *PAL* gene transcripts was found to be highest in the months of October and November in all the three *Ocimum* species. In *O. sanctum*, the expression was *PAL* transcripts was highest in the month of November while in *O. kilimandscharicum* it was highest in October and in *O. gratissimum* the expression was nearly equal in October and November. In the month of December, the transcripts of *PAL* specifically decreased in *O. sanctum* and *O. kilimandscharicum* while there was not much decrease in the *O. gratissimum*’s *PAL* gene expression. The expression of *C4H* gene showed quite a uniform pattern from July to December. *C4H* gene was found to be expressed highest in *O. sanctum* in the month of November. The expression of *C4H* in *O. gratissimum* was comparable with that of its expression in *O. sanctum* and was highest in the month of October. The expression pattern of *C4H* transcripts in all the three species of *Ocimum* was more or less consistent. The *4CL* transcript expression was found to be highest in the months of October and November. In the present study, highest *4CL* transcript expressing species was *O. gratissimum* in the month of October. In *O. sanctum* as well as the *O. gratissimum* expression pattern of 4CL gradually increased from July to November, but in case of *O. sanctum* got decreased in December with not much effect in *O. gratissimum*. The expression of *C3H* transcript was found to be throughout from the month of July upto November significantly dropped down in December in all the *Ocimum* species in the present study. *O. sanctum* showed the highest expression of *C3H* transcript followed by very low expression *O. kilimandscharicum* and relatively very low in *O. gratissimum. C3H* transcript was among the lowest expressing one in *O. gratissimum* which had maximum high expressing phenylpropanoid gene transcripts. The expression pattern of *C3H* transcripts in *O. sanctum* and *O. kilimandscharicum* was analogous to *C4H* with an exception of *O. gratissimum. O. gratissimum* showed the highest expression of *COMT* gene transcript. The expression was by far highest during the month of July and fairly in August, September, October and November, but gradually decreases during December. Expression pattern of *COMT* transcript in *O. sanctum* was also comparable to *O. gratissimum* while *O. kilimandscharicum* showed very low expression of transcript. The expression of *CCR* gene transcript was found to be highest in *O. kilimandscharicum* which was comparable to *O. gratissimum* and was least in *O. sanctum. CCR* expression first increase upto October in *O. kilimandscharicum* and decreased thereupon, but the decrease was not very significant. However, the expression pattern of *CCR* transcript in O*. gratissimum* was more or less constant while showed an increasing pattern in case of *O. sanctum*. The *CAD* gene transcript expression was almost similar and uniform in all the three *Ocimum* species throughout all the six months with an exceptionally high expression observed in *O. gratissimum* during the month of July. *O. gratissimum* showed the highest expression of the *EGS* gene transcript with a gradual increase of expression from July and upto highest in the month of November, thereafter decreasing in December. *O. sanctum* followed *O. gratissimum* in *EGS* transcript expression but in the similar temporal series. *O. kilimandscharicum* was found to be having very less expression of *EGS* gene transcript as compared to the other two *Ocimum* species. The expression of *EOMT* gene transcript was exceptionally high in *O. sanctum*, found to be increasing from the month July to November. The highest expression of the transcript was found to be observed during the month of November and thereafter decreases, onwards December. The other two *Ocimum* species showed the expression of *EOMT* transcript in very trace levels.

### Expression profiling of secondary metabolism related transcription factors (TFs)

**Figure 5:**
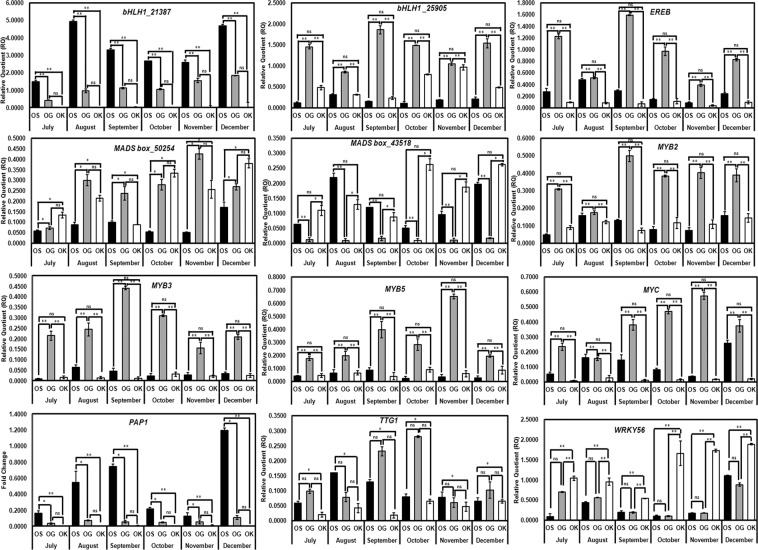
Relative expression levels of bHLH_21387, bHLH_25905, EREB, MADS box_50254, MADS box_43518, MYB2, MYB3, MYB5, MYC, PAP1, TTG1 and WRKY56 transcription factors in *O. sanctum* (OS)*, O. gratissimum* (OG) and *O. kilimandscharicum* (OK) during six months starting from July’ 2018 till December’ 2018 determined by Real-time RT-PCR. Data are means ± SD (for at least three replicates), and the y-axis represents the relative quotient (RQ). Statistical analysis was performed using HSD tukey test, standard weighted-means analysis at *P < 0.01 and **P < 0.05 levels of significance while ‘ns’ means non-significant.

Expression pattern of transcription factors pertaining to the secondary metabolism were also studied in the present investigation. Based on the equivalent transcript abundance of digital gene expression data of prior transcriptome analysis^39^ comparing *O. sanctum* and *O. basilicum*, some of the TFs involved in the secondary metabolism were sorted for present analysis. The TFs selected for the expression studies were- *bHLH1*, *EREB*, *MADS box*, *MYB2, MYB3, MYB5, MYC, PAP1*, and *TTG1*. Analysis was carried out using the primers designed from the comparative transcriptome sequencing data of *O. sanctum* and *O. basilicum* reported recently by Rastogi *et al*.^39^ (Supplementary Table 2). Only primer sequence of TF *WRKY* was designed from the *O. basilicum* ‘TrichOME Database’ (http://www.planttrichome.org/trichomedb/estbyspecies_detail.jsp?species=Ocimum%20basilicum) (Supplementary Table 2) because the annotations were lacking *WRKY* which is considered to be an important TF involved in regulation of secondary metabolism pathways^42^. In case of *bHLH1* and *MADS BOX* TFs the two- two transcripts were selected as these transcript sequences were non-overlapping and were showing equivalent transcript abundance in digital gene expression data of *O. sanctum* and *O. basilicum* leaf transcriptomes. As evident from the Fig. 5, out of the 12 TFs selected in the present study, the maximum number of TFs i.e., 8 was expressed higher in *O. gratissimum* followed by *O. sanctum* and *O. kilimandscharicum*. Now considering the expression pattern of each TF it was observed that two non- overlapping transcripts of *bHLH1* (*bHLH1_21387* and *bHLH1_25905*) showed an entirely different pattern of expression with respect to each other. Expression of *bHLH1_21387* was found to be highest in *O. sanctum* but there was no temporal pattern of expression. Expression of *bHLH1_21387* in *O. sanctum* increased from July to August, gradually decreased in September with nearly no change in expression during October and November and a sudden increase in the month of December. The expression of *bHLH1_21387* in *O. sanctum* during the December month was comparable to its expression in August. On the contrary, expression of *bHLH1_25905* was highest in *O. gratissimum* but a specific temporal pattern of expression was also not observed, while in case of *O. kilimandscharicum* there was a gradual increase in *bHLH1_25905* transcript expression from the month of July till November and got decreased December onwards. *O. sanctum* showed the lowest expression of *bHLH1_25905* transcript and also not much of significant difference was seen in the expression pattern in all the six months. Observing the expression pattern of *EREB* transcript it was found to be expressing highest in *O. gratissimum* during the month of September and decreased thereafter. In case of *O. sanctum EREB* expression increased from July to August which also got decreased upon maturity, while in *O. kilimandscharicum* there was a uniform expression pattern of this transcript throughout all the six months. Considering the expression pattern of another non-overlapping TF transcripts *MADS box_50254* and *MADS box_43518* contrasting results were obtained. In case of *MADS box_50254* transcript, expression was highest in *O. gratissimum* which gradually increased from July upto highest in November and decreased in December. Antagonistically, expression of *MADS box_50254* got increased from July upto the month of December in both *O. kilimandscharicum* and *O. sanctum*. Interestingly, *MADS box_43518* transcript showed the similar expression pattern as of *MADS box_50254* in case of *O. kilimandscharicum* and *O. sanctum*, while a uniform expression pattern but very low transcript abundance of *MADS box_43518* was observed in *O. gratissimum*. Analyzing the expression pattern of the three transcripts of MYB TF family members, *MYB2, MYB3* and *MYB5*, it was observed that expression of all the three MYB transcripts was highest in *O. gratissimum* and more or less equivalent pattern in *O. kilimandscharicum* and *O. sanctum*. Expression of *MYB3* and *MYB5* transcripts in *O. gratissimum* increased from young stage (July) and then decreased upon maturity (December) while *MYB2* expression increased from July to September and was nearly constant in the successive months upto December. However, the expression pattern of the three *MYB* family TFs in *O. kilimandscharicum* and *O. sanctum* was nearly temporally unaffected. Next, the *MYC* transcript expression was also found to be highest in *O. gratissimum* which increased from July upto November and got reduced in December, while its expression increased in case of *O. sanctum* from July to December and was least as well as unaffected in *O. kilimandscharicum*. Expression study data of *PAP1* TF transcript reveals *O. sanctum* to be highest *PAP1* expressing *Ocimum* species among the three whose expression increases as the plant matures and found to be highest in during the month of December. *PAP1* expression in other two *Ocimum* species, *O. gratissimum* and *O. kilimandscharicum* was very low as compared to *O. sanctum* and also no noticeable temporal effect was observed in *PAP1* expression pattern as there laid almost evenly distributed transcript abundance throughout the six months. A canonical temporal expression pattern of *TTG1* TF transcript was observed in case of *O. gratissimum* and *O. sanctum* while in case of *O. kilimandscharicum* the expression increased from July to October and thereafter remained constant in November and December. The order of expression of *TTG1* was highest in *O. gratissimum* followed by *O. sanctum* and then *O. kilimandscharicum*. The last studied TF for expression study was *WRKY56* which demonstrated the same expression pattern in all the three *Ocimum* species i.e., the expression increases as the plant ages. The highest expression was observed in *O. kilimandscharicum* followed by the other two, *O. gratissimum* and *O. sanctum*.

### Enzyme activity of some important phenylpropanoid pathway enzymes

**Figure 6:**
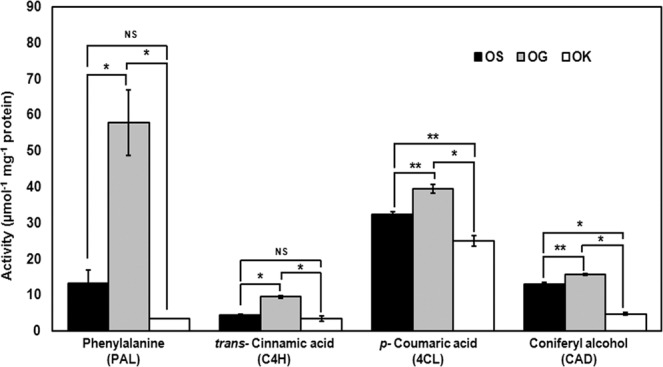
Enzyme activity of PAL, C4H, CAD quantified in terms of product formed and of 4CL in terms of substrate utilized represented per mg of the total plant protein from *O. sanctum* (OS)*, O. gratissimum* (OG) and *O. kilimandscharicum* (OK) at maturity. L-Phenylalanine, *trans*- cinnamic acid, *p*- coumaric acid and coniferyl alcohol were used as the substrates for PAL, C4H, 4CL and CAD, respectively. Activities are given as means of two independent assays ± SD (standard deviation). Statistical analysis was performed using HSD tukey test, standard weighted-means analysis at *P < 0.01 and **P < 0.05 levels of significance while ‘ns’ means non-significant.

Crude protein extracts obtained from young leaves of the mature plant were assayed for activity for the key phenylpropanoid pathway enzymes in leading to the phenylpropenes, as well as for an intermediate enzyme that might also be involved in phenylpropenes biosynthesis. These enzymes included phenylalanine ammonia lyase (PAL), cinnamate 4-hydroxylase (C4H), 4-coumarate: CoA ligase (4CL), and cinnamyl alcohol dehydrogenase (CAD). Figure 6 shows the activity of the enzymes per mg protein. The order of activity for the four enzymes in the three *Ocimum* species was highest in *O. gratissimum* followed by *O. sanctum* and least in *O. kilimandscharicum*.

### Total phenolic content, anthocyanin content and chlorophyll content

Total phenolics, anthocyanin and chlorophyll contents were estimated from the leaves of the mature plant of all the three *Ocimum* species in order to know their variability in terms of these three constituents in order to get some correlation of the these constituents composition with that of the gene expression profiling of phenylpropanoid pathway as well as transcription factors.

**Figure 7:**
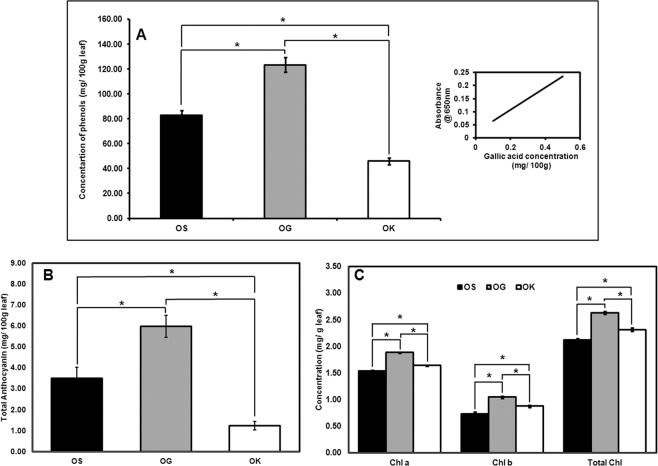
The content of (**A**) total phenols, (**B**) anthocyanin and, (**C**) chlorophyll determined in mature plant for comparing the three *Ocimum* species, *O. sanctum* (OS)*, O. gratissimum* (OG) and *O. kilimandscharicum* (OK). Data are means ± SD (for at least three replicates). Statistical analysis was performed using HSD tukey test, standard weighted-means analysis at *P < 0.01 and **P < 0.05 levels of significance.

The phenolics and the pigment analysis results as shown in the Fig. 7 proved *O. gratissimum* to be richest of all, the total phenolics, anthocyanin as well as chlorophyll pigments.

## Discussion

On comparing the oil yields and the trichome densities of the three *Ocimum* species, it was observed that as the trichome density decreases upon the plant maturity, the oil yield also decreases (Table 1). The essential oil profiling showed that both *O. sanctum* and *O. gratissimum* were rich in eugenol which is a phenylpropene compound whereas *O. kilimandscharicum* was rich in camphor, a well-known terpenoid (Fig. 1 and Supplementary Table 3). As also reported by Joshi^43^ eugenol was the principal constituent of *O. gratissimum* and *O. sanctum*^44^ while camphor in case of *O. kilimandscharicum*^45^. Oil yield of *O. gratissimum* (1.3%) was highest followed by *O. sanctum* (0.87%) and *O. kilimandscharicum* (0.73%) and similar was the pattern of peltate glandular trichome densities (Fig. 2) except in *O. kilimandscharicum. O. kilimandscharicum* did not show much difference in the trichome distribution throughout the six months and also had the least number of trichomes per mm^2^ leaf area and also least oil yield as compared to *O. sanctum* and *O. gratissimum*. Since the apical leaves were used for the trichome density studies, the number of trichomes increased upto the optimum metabolite expression stage (July to October) and thereafter the number decreased to leaf expansion. While studying the changes in leaf trichomes and epicuticular flavonoids during leaf development in birch taxa, Valkama *et al*.^46^ also concluded that rapid decline in the density of leaf trichomes due to growth dilution in expanding leaves, as the total number of trichomes per leaf remained constant and neither the development nor shedding of trichomes at later growth stage occurs. In another study, Adebooye *et al*.^47^ also described that the morphology and density of trichomes and stomata of *Trichosanthes cucumerina* also got affected by leaf age, densities decrease as leaf age increases. Tozin *et al*.^48^ while analyzing the density of glandular trichome density and essential oil profile of inflorsences and leaves of *Lippia origanoides* Kunth belonging to family verbenaceae also reported that higher essential oil yield in the inflorescences as compared to the leaves. In a separate investigation by Werker^49^, it was demonstrated that trichomes remain functional in mature leaves, contrastingly Gairola *et al*.^50^ also reported that at leaf maturity, the functional role of trichomes becomes less important and they therefore senesce or wither. All of the differences in the trichome densities of the three *Ocimum* species could be related to genetic or physiological or evolutionary mechanisms operating within the genus, which need further investigation. Essential oil of the genus *Ocimum* is a secondary metabolites reservoir and has been suggested to be correlated with the chromosome numbers of species as well as the oil yield^39,51^. The chromosome numbers of *O. sanctum* var. CIM Ayu (2n = 16), *O. gratissimum* is higher (2n = 40) and *O. kilimandscharicum* (2n = 38)^39,52,53^ also support the earlier report of correlation between essential oil yield and chromosome number.

In secondary metabolism, phenylpropanoid biosynthesis is one of the most important pathways as it leads to synthesis of a large group of natural products^22,54^. The core phenylpropanoid pathway involves three enzymes, PAL, C4H, and 4CL. PAL is the first enzyme in the pathway that catalyzes the conversion of L -phenylalanine to *trans*-cinnamic acid. Subsequently, C4H, which is a member of cytochrome P450 super-family, hydroxylates *trans*-cinnamic acid into *para*-coumaric acid. Lastly, the formation of *p*-coumaroyl CoA from *p*-coumaric acid takes place by the reaction catalyzed by 4CL leading to the production of hydroxycinnamic acids, monolignols/lignin, coumarins, benzoic acids, stilbenes, anthocyanins and flavonoids^22,55^. In *Ocimum* species, the phenylpropanoid pathway is an important one as it leads to the synthesis of many commercially important phenylpropenes like- eugenol, methyleugenol, chavicol, methylchavicol in the leaf essential oil^38,39,44,56^. The phenylpropenes synthesized in plant aerial parts helps in plant defense against herbivores and pathogens^56^ and are also imperative in human diet^57,58^. Biosynthesis of these phenylpropenes is localized in the specialized glands known as glandular trichomes on the surface of leaves^3,56^.

PAL is an important enzyme as it is a link between primary and secondary metabolism. It is also a key regulatory enzyme in the phenolics biosynthesis^59^ as high activity of PAL is usually associated with the accumulation of phenolic compounds in fruit tissues of several species^60^. Highest expression of *PAL* (Fig. 4) also correlated with the highest enzyme activity (Fig. 6), highest total phenol and anthocyanin contents (Fig. 7A,B) in *O. gratissimum* followed by *O. sanctum* and *O. kilimandscharicum*. Expression of the *PAL, C4H* and *4CL* transcripts in all the three *Ocimum* species first increased and then decreased as the plant attained maturity. The expression was optimal at the duration of high oil yield respective to each of the three *Ocimum* species. Xu *et al*.^61^ have also proven that the expression level of *GbPAL* from *Ginkgo biloba* was lowest at the beginning of leaf growth, increased gradually, decreased thereafter and subsequently increased further and then remained relatively constant. The results obtained at the metabolic, transcript and protein level support the hypothesis^62^ that the PAL, C4H, and 4CL genes or gene families irrespective of their variable sizes, represent a case of tight regulation, possibly mediated through large structural and functional similarity in TF binding with their promoters^63–66^. The real-time expression results of the seven TFs (*bHLH1_25905, EREB, MADS box_50254, MYB3, MYB5, MYC and TTG1*) out of twelve TFs studied (Fig. 5) in the present investigation also show a similar expression pattern as of the *PAL, C4H* and *4CL* transcripts in the three *Ocimum* species are also in coherence with the above hypothesis. As also suggested by Koopmann *et al*.^67^, the results of the present study indicate to the possibility of the formation of a true multienzyme complex by PAL, C4H and 4CL enzymes.

The activity of C3H in biosynthesis of lignin and many other phenylpropanoid pathway products in plants has been well documented, however, conditions suitable for assay of the enzyme explicitly, still remain unclear. Although *p*-coumarate acts as the substrate of C3H but its significant activity towards other *para* hydroxylated substrates cannot be ignored^67^. Franke *et al*.^68^ in their work revealed CYP98A3 is encoded by *REF8* gene which is required for the synthesis of wild-type lignin precursors and sinapate esters in *Arabidopsis*. Gang *et al*.^69^ have reported that the differential production of *meta* hydroxylated phenylpropanoids in sweet basil is controlled by the activities of specific acyltransferases and hydroxylases found in the peltate glandular trichomes and leaves. In the present investigation, the expression pattern of *C3H* transcript in the three *Ocimum* species did not show a specific trend. In case of *O. sanctum* and *O. gratissimum*, the expression first increased, then decreased, again increased and finally decreased while it was nearly constant in case of *O. kilimandscharicum* (Fig. 4). Till date the activity of C3H was considered essential for the lignin biosynthesis in plants^70,71^ but the possibilities of synthesizing other compounds may not be overlooked. As also reported in our previous work^38^ a new function of *4CL* gene was explored towards eugenol biosynthesis rather than considering its conventional involvement in lignin biosynthesis. Hence, a further investigation is required to finally prove its role in the synthesis and/or regulation of phenylpropenes biosynthesis and to evidence the highest transcript expression in *O. sanctum*. Expression of next gene transcript, *COMT* was found to be decreased as the plant attained maturity in case of *O. gratissimum* and *O. sanctum* whereas in *O. kilimandscharicum* there was no major change in expression pattern was observed (Fig. 4). It has already been discussed that there is decrease in essential oil metabolites as the plant attains maturity. This nature of expression of *COMT* gene transcript expression may be elucidated by the experiments carried out by Gang *et al*.^56^ where the role of *COMT* in phenylpropene biosynthesis was evident when the northern blot showed high expression of *COMT* in glandular trichomes as compared to the whole leaf while studying the relative abundance of mRNA in the peltate glandular trichomes.

The activity of CCR and CAD enzymes till date has been attributed to lignin biosynthesis^72–74^. The *CCR* and *CAD* gene encode the enzymes which catalyze the first and last steps of lignin monomer biosynthesis, respectively and are closely related members of the short-chain dehydrogenase/reductase (SDR) superfamily^75^. Thus, the constant and increasing patterns of *CCR* and *CAD* transcripts expression in the all the three *Ocimum* species could be correlated with the plant aging (Fig. 4).

EGS and EOMT are the terminal genes which encode for the enzymes responsible for the synthesis of eugenol and methyleugenol from the coniferyl acetate and eugenol as the substrates, respectively^76^ as also shown in Fig. 3. Expression of *EGS* transcript was found to be expressed highest in *O. gratissimum* followed by *O. sanctum* and vice versa in case of *EOMT* expression pattern while, *O. kilimandscharicum* showed very low expression of *EGS* and even lesser expression of *EOMT* transcript (Fig. 4). As evident from the Fig. 1, *O. gratissimum* and *O. sanctum* had high percentages of eugenol content in their leaf essential oil; hence the high expression of *EGS* in both the two species gets justified. Gang *et al*.^77^ while characterizing the phenylpropene *O*-methyltransferases from sweet basil with 13 phenolic acid substrates demonstrated that EOMT1 enzyme gave 100% activity with eugenol as a substrate, simultaneously it also gave 29%, 26% and 24% activities with guaicol, isoeugenol and chavicol as substrates. Essential oil profile of *O. sanctum* and *O. gratissimum* (Supplementary Table 3) also confirms the eugenol and iso-eugenol presence in high percentages and hence, the high expression of these two gene transcripts in the two *Ocimum* species may be correlated. Since these two enzymes, EGS and EOMT are localized in the glandular trichomes of leaf, the expression of the transcript decreases as the leaf expands upon maturity.

Transcription factors (TFs) play a dominant role in gene regulation of all plant growth and development aspects, inclusive of secondary metabolism. Recent years, have added to the number of transcription factors involved in plant secondary metabolism regulation. However, the possibility of existence of other mechanisms regulating specific pathways cannot be overruled. Several families of TFs have been ascribed to be regulators of plant secondary metabolism but a few important ones with equivalent digital gene expression from the comparative *O. sanctum* and *O. basilicum* transcriptome sequencing data^39^ were sorted for the temporal expression studies of three *Ocimum* species. These include two non-overlapping transcripts of *bHLH1* (*bHLH_21387* and *bHLH_25905*), *MADS box* (*MADS box_50254* and *MADS box_43518*) each and single transcripts of *EREB, MYB2, MYB3, MYB5, MYC, PAP1*, and *TTG1* (Fig. 5). MADS box proteins, the MYB and bHLH (basic-helix-loop-helix) families have significantly expanded in the past 100–600 million years and are extensively reviewed^78^. TFs generally form complexes in order to regulate the metabolic pathways as evident by several examples. It has been reported that the *MYB* and *bHLH* TFs function cooperatively and flavonoid biosynthesis is one of the best best-studied pathway of the combinatorial gene regulation by interactions between the two^79^. Gonzalez *et al*.^80^ demonstrated the regulation of the anthocyanin biosynthetic pathway by transcriptional complex formation of *TTG1* (transparent testa glabra1)/ *bHLH*/ *MYB* TFs in *Arabidopsis* seedlings. Not only was this TF complex, *MYC* also suggested to be involved in regulation of anthocyanin biosynthesis in *Perilla frutescens*^81^, a member of the same lamiaceae family to which *Ocimum* belongs. Considering the diverse functions of these TFs it becomes extremely difficult to give explanations to the intricate role of *bHLH, MYB, MYC* and *TTG1* TFs. PAP1 (production of anthocyanin pigment1) being well described for its involvement in the anthocyanin biosynthetic pathway^82^ but Sekhon *et al*.^83^ and Pourtau *et al*.^84^ have also reported *PAP1* and *PAP2* to be involved in senescence induced by pollination prevention in maize and sugar application in *Arabidopsis*, respectively. Hence, the expression pattern of *PAP1* in *O. sanctum* may be due to some stress induced at the onset of winter season in November and December as *Ocimum* is a plant of tropics. But no specific trends in the expression pattern of *O. gratissimum* and *O. kilimandscharicum* were observed which might be due to the fact that *O. sanctum* might be comparatively more susceptible to cold climate which needs experimental confirmation. Contrastingly, MADS which is an acronym for the four founder proteins MCM1 (from *Saccharomyces cerevisiae*), AGAMOUS (from *Arabidopsis*), DEFICIENS (from *Antirrhinum*), and SRF (a human protein), on which the definition of this gene family is based. The network of these *MADS box* genes is not only imperative in contributing to floral organ identity but also in floral meristem identity^85^. The increasing temporal pattern of expression of *MADS box* gene transcript in the three *Ocimum* species of the present study supports the fact of involvement of *MADS box* gene in flower organ development as meristem. The ERF (ethylene response factor) family formerly known as EREBP (ethylene-responsive element binding proteins) is attributed to regulation of biological processes related to plant growth, metabolism, development, and response to abiotic and biotic stresses^86^. Since the *EREB* transcript reveals an increasing and further decreasing temporal expression pattern in the three *Ocimum* species under the investigation, it might be involved in some of the biological process active during the plant development and slows down as the plant attains maturity. WRKY proteins comprise a large family of TFs which imperative to developmental and defense response in plants, hence the higher expression of *WRKY* transcript in the three *Ocimum* species towards the plant maturity might be due to the plant response against the abiotic stresses. The phenylpropenes found in glandular trichomes of *Ocimum basilicum* play an important in plant resistance against herbivores^56^. Simultaneously, Valkama *et al*.^46^ suggested that during the birch leaf development, the amount of osmiophilic material (phenolics containing *o*-dihydroxy groups) declines, however 20–40% of cells in aged trichomes possess it.

This study provides a comparative description in trichome number and expression pattern of important genes of phenylpropanoid biosynthesis pathway as well as the transcription factors involved in the secondary metabolism with respect to the differential accumulation and regulation of essential oil metabolites and their composition among three *Ocimum* species. The final number of trichomes is *Ocimum* leaf is ascertained at the young stage and does not change during leaf development. On the contrary, the trichome density as well as phenylpropens tends to decline with leaf age. Since the basils are susceptible to winter season, the expression pattern of the genes and transcription factors discussed herewith may be due to some abiotic (cold) or biotic (insects, fungal pathogens etc.) which often attack the plant during this season. A very scarce literature is available to infer the interaction of such abiotic and biotic plant stress exerted over the plant. Present investigation in light of trichomes as well as gene expression studies could be exploited for genetically improving the essential oil biosynthesis in *Ocimum* species which are becoming highly desirable for fragrance, flavor and pharmaceutical industries.

## Methods

### Plant material

Leaf tissues from the 3 month plants after seed sowing [*O. gratissimum*, var: CIM-Ayu of *O. sanctum* L^44^., and *O. kilimandscharicum*] were collected monthly in the year 2018 in triplicates from the research field at the CSIR-Central Institute of Medicinal and Aromatic from July till December.

### Extraction and analysis of essential oil

Hydro-distillation of collected plant leaves was conducted in a Clevenger-type apparatus for two hours. 1 µl of 1:10 pentane diluted essential oil was injected in GC-MS (Agilent Technologies 7980 A gas chromatograph system with the 5977 A mass selective detector) for analysis. The HP5-MS column with dimension 30 m × 250 µm having film thickness 0.25 µm was used for obtaining the peak separation in the chromatogram. Helium in a split ratio of 10:1 and flow rate of 1 ml/min was used as the carrier gas. The running condition for the samples was 40° for 5 min as initial hold, subsequently 150 °C at the flow rate of 3 °C/min, followed by a ramping of 5 °C/min until the temperature reaches 200 °C and finally a hold for 10 min after the temperature reaches 300 °C with a ramp rate of 10 °C/min. Mass spectrometry was conducted at 230 °C as a transfer line and ion source temperature while, 150 °C as quadrupole temperature, 70 eV ionization potential and 50 to 550 atomic mass units scan range. Version 2.0 g of NIST/EPA/NIH mass spectral library was used for compound identification (Agilent Technologies, Palo Alto, CA, USA). The relative abundance of particular constituent was considered as the area percent.

### Analysis of trichome density

Microscopic analysis was carried out from the leaves of fourth nodes of the plant shoot-tips. Trichomes on the leaves were photographed under an inverted fluorescence microscope (Nikon Eclipse Ti-S) on 20 × magnification with auto exposure of 15 milliseconds and analog gain of 2.0X at a 100 µm scale bar. Thereafter all the images captured were examined with the help of NIS- Elements F (version 4.0) imaging software for the estimation of trichome density as the total number of trichomes within an area of 1.0 mm^2^ on the leaf abaxial side in a sample size of 10 plants per month from each of the three *Ocimum* species. Significant differences among populations between months of the collection were calculated using Tukey–HSD post hoc test at *p* > 0.01, *p* > 0.05, *p* > 0.1.

### Trichome and RNA isolation

Young leaves of all the three *Ocimum* species (*O. sanctum, O. gratissimum* and *O. kilimandscharicum*) were used to isolate the glandular trichomes following the method used by Rastogi *et al*.^38^. The total RNA was isolated from the isolated glandular trichomes using Spectrum Plant Total RNA Kit (Sigma). 2 µg of total RNA was used for the cDNA synthesis *via* Revert Aid Premium First Strand cDNA Synthesis Kit (Thermo).

### Quantitative RT–PCR analysis

Quantitative realtime PCR was performed by the protocol given by Rastogi *et al*.^38^ which utilized SYBR Green chemistry (Thermo). The Primer Express Software version 2.0 (Applied Biosystems) was used for the designing of gene-specific primers and were ordered from Integrated DNA Technologies, India (Supplementary Table 1 and 2). The experiment was conducted in ‘7900HT Fast Real Time PCR System’ (Applied Biosystems) with five biological replicates, and the reaction specificity was evaluated by analyzing the melting curve. The parameters of the thermal cycling were: 50 °C for 2 min (initial hold); 95 °C for 10 min (initial denaturation); and 40 amplification cycles (95 °C for 15 s; and 60 °C for 1 min). Subsequently additional steps (60 °C for 15 s, 95 °C for 15 s and 37 °C for 2 min) were followed to get the dissociation curve. Actin of *O. sanctum* (details provided in the Supplementary Table 1) was used as an endogeneous control to quantify the relative mRNA levels^38,87^. ∆∆C_t_ method was used for relative quantification of gene transcripts through Sequence Detection System (SDS) software version 2.2.1. As a result of real-time PCR, the C_t_ (threshold cycle) values thus obtained were used to calculate ∆C_t_ value (target-endogenous control). Thereafter, ∆∆C_t_ was calculated for the quantification by determining the fold difference in gene expression [∆Ct target – ∆Ct calibrator]. Finally, 2^−∆∆CT^ was determined as relative quotient (RQ).

### Total phenols, anthocyanin chlorophyll estimation

Anthocyanin content was estimated following the protocol of Neff and Chory^88^. Anthocyanin quantification was carried out by incubating 1 g leaf samples (grounded in liquid nitrogen) overnight in 150 ml of with 1% HCl acidified methanol in triplicates. Further 100 ml of distilled water and 250 ml chloroform was added to separate anthocyanins from chlorophylls. Absorbance at 530 nm and 657 nm were recorded to determine total anthocyanins using a spectrophotometer (Elico). Relative amount of anthocyanin per gram leaf sample was calculated by subtraction of absorbance at 657 nm from the absorbance at 530 nm.

Total phenolic content was determined by Folin- Ciocalteu method using gallic acid as phenolic standard^89^. About 100 mg powdered leaf samples of *O. sanctum*, *O. kilimandscharium* and *O. gratissimum* were extracted with 0.5 µl of 80% ethanol in triplicates. The extract was centrifuged for 20 min and the supernatant was collected. The supernatants were evaporated to dryness and dissolved in 0.5 ul of water. Different aliquots of the dissolved extracts were pipetted (2–20 µl) into the micro-centrifuge tubes. The volume of each micro-centrifuge tube was made up to 300 µl final volume with the double distilled water. About 50 µl of Folin-Ciocalteau reagent was added into each tube. After 3 min, 200 µl of 20% Na_2_CO_3_ solution was added into the each tube and mixed thoroughly. Each tube was now placed in boiling water for exactly one min. The samples were cooled and measured at 650 nm absorbance using micro-titer plate. The concentration of total phenols was estimated using standard curve and expressed as mg phenols/100 g materials.

Chlorophyll extraction was performed by the protocol given by Sadasivam and Manickam^89^. About 1 g powdered leaf samples of *O. sanctum*, *O. kilimandscharium* and *O. gratissimum* were extracted with 80% chilled acetone in triplicates till the residues turned colorless. The supernatant was collected into the volumetric flask and the final volume was made upto 100 ml with 80% of chilled acetone. The extracted solutions were measured as 645 nm, 663 nm and 652 nm absorption against 80% acetone as blank. The amount of chlorophyll present in the extract mg chlorophyll per g tissue was calculated using the following equations:

$$\begin{array}{rcl}{\rm{mg}}\,{\rm{chlorophyll}}\,{\rm{a}}/{\rm{g}}\,{\rm{tissue}} & = & 12.7({{\rm{A}}}_{663})-2.69({{\rm{A}}}_{645})\times {\rm{V}}/1000\times {\rm{W}}\\ {\rm{mg}}\,{\rm{chlorophyll}}\,{\rm{b}}/{\rm{g}}\,{\rm{tissue}} & = & 22.9({{\rm{A}}}_{645})-4.68({{\rm{A}}}_{663})\times {\rm{V}}/1000\times {\rm{ > W}}\,and\\ {\rm{mg}}\,{\rm{total}}\,{\rm{chlorophyll}}/{\rm{g}}\,{\rm{tissue}} & = & 20.2({{\rm{A}}}_{645})+8.02\,({{\rm{A}}}_{663})\times {\rm{V}}/1000\,\times {\rm{W}}\end{array}$$

where, A = absorbance at specific wavelengths,

V = final volume of chlorophyll extract in 80% acetone

W = fresh weight of tissue extracted

### Enzyme assays

Young leaves were used to prepare the soluble protein extracts. Whole leaves of individual species were weighed and grinded in liquid nitrogen in triplicates. The extraction was carried out in ice-chilled protein extraction buffer (10:1, w/v), containing 50 mM BisTris [2-[bis(hydroxyethyl)amino]-2-(hydroxymethyl)-1-propane- 1,3-diol] HCl, pH 8.0, 14 mM β-mercaptoethanol, and 10% (w/v) glycerol followed by an incubation of 30 min on ice. The ground mixture was then centrifuged at 4 °C for 20 min at 14,000 g to get the protein extract as a clarified supernatant which was transferred to a new tube. The Bradford method^90^ was used to quantify the concentration of protein in the extract. The protein thus isolated was used for the assay of PAL (phenylalanine ammonia lyase), C4H (cinnamate-4-hydroxylase), 4CL (4-coumarate: CoA ligase) and CAD (cinnamyl alcohol dehydrogenase) enzymes.

PAL, C4H, 4CL, and CAD activities were measured following the procedures described by Gang *et al*.^56^, Misra *et al*.^91^, Rastogi *et al*.^38^, Fu *et al*.^74^, respectively. Enzyme assay for each enzyme was set in reaction volume of 1 ml containing 1 mg of the plant protein extracted. There were two controls taken for this enzyme assay, one included all reaction components except the protein and another had all reaction components except substrate. Reactions were incubated for 2 hours at 30 °C and after that it was ended by the addition of 50 µl 6 N HCl. The product was extracted twice by adding equal volume of ethylacetate, vortexing, and centrifuging at 14,000 g for 5 min, followed by evaporation of organic phase in vacuum. Product identification was verified by gradient high-performance liquid chromatography (HPLC) (LCMS-2010 EV, Shimadzu) as described by Proestos and Komaitis^92^. The mobile phase consisted of eluent A [2% (*v/v*) acetic acid/water] and eluent B [methanol: acetonitrile (50:50 *v/v*)]. Extracts were separated on Symmetry C-18 column (5 µm, 4.6 × 250 mm) using a gradient program: 10% B (0–5 min.), 40% B (5–25 min.), 45% B (25–35 min.), 55% B (35–40 min.) and finally 10% B (40–45 min.) at the flow rate of 1 ml/min. Column effluent was monitored at wavelengths of 254 nm, 280 nm, 320 nm and the product was recognized by spectral scans using the photodiode-array detector followed by comparing retention time and UV spectrum with that of genuine standards. In case of 4CL enzyme assay, the activity was measured in terms of substrate utilization rather than product formation due to the commercial unavailability of *p*-coumaroyl CoA standard.

### Statistical analysis

One‐Way Analysis of Variance (ANalysis Of VAriance) with post-hoc Tukey HSD (Honestly Significant Difference) test^93^ was used for performing all the statistical analysis used in the study at *P < 0.01 and **P < 0.05 levels of significance with ‘ns’ meaning non-significant.

## Supplementary information

Supplementary information.
