# Supplementary material for: Comparative temporal metabolomics studies to investigate interspecies variation in three Ocimum species
Source: Sci Rep. 2020 Mar 23;10:5234. doi: 10.1038/s41598-020-61957-5 (PMC7089951; doi:10.1038/s41598-020-61957-5)
Supplement: Supplementary file 1 — Supplementary information. [file 41598_2020_61957_MOESM1_ESM.pdf]

Comparative temporal metabolomics studies to investigate interspecies variation in three *Ocimum* species

Shubhra Rastogi[1], Saumya Shah[2], Ritesh Kumar[2], Ajay Kumar[2], Ajit Kumar Shasany[2]*

[1]Centre for Biotechnology, Shiksha 'O' Anusandhan University, Bhubaneswar- 751003, Odisha, India

[2]Biotechnology Division, CSIR- Central Institute of Medicinal and Aromatic Plants, PO CIMAP, Lucknow-226015, UP, India

*Corresponding author e-mail:

ak.shasany@cimap.res.in

SUPPLEMENTARY INFORMATION

Supplementary table 1. Details of primer sequences used for qRT-PCR analysis of phenylpropanoid pathway genes.

S. No.	Name of primers	Primer Sequence (5' – 3')	Details
1	Actin_SyF	AGATTCCTCCAGCAAATCTTTCTC	Forward primer for Actin designed from SRA study acc. no. SRP039008 (transcript id: Locus_4043_Transcript_3/5_Confidence_0.368_Length_944)
2	Actin_SyR	CTTTCTGGTGGAACAGCATCAA	Reverse primer for Actin designed from SRA study acc. no. SRP039008 (transcript id: Locus_4043_Transcript_3/5_Confidence_0.368_Length_944)
3	PAL_SyF	TCCCAACGAGTTCACATCTTGAT	Forward primer for PAL designed from SRA study acc. no. SRP039008 (transcript id: Locus_19788_Transcript_1/1_Confidence_1.000_Length_2482)
4	PAL_SyR	TTGGCTAATCCCGTGACTA ACC	Reverse primer for PAL designed from SRA study acc. no. SRP039008 (transcript id: Locus_19788_Transcript_1/1_Confidence_1.000_Length_2482)
5	C4H_SyF	TTGGTCCGGGAGTCCAAATA	Forward primer for C4H designed from SRA study acc. no. SRP039008 (transcript id: Locus_2389_Transcript_3/4_Confidence_0.600_Length_2035)
6	C4H_SyR	TCACGGCCTGAAGGTATGG	Reverse primer for C4H designed from SRA study acc. no. SRP039008 (transcript id:Locus_2389_Transcript_3/4_Confidence_0.600_Length_2035)
7	4CL_SyF	AAGAAGCTGTCAAGGAATTCATTTC	Forward primer for 4CL designed from accession no. HM990148
8	4CL_SyR	GGGAATGGCATGGACAAAGA	Reverse primer for 4CL designed from accession no. HM990148
9	C3H_SyF	AGGGATGGCCAGGATCTGA	Forward primer for C3H designed from accession no. HM990156
10	C3H_SyR	ACACACTTTTCTCACCTTGACGTAGT	Reverse primer for C3H designed from accession no. HM990156
11	COMT_SyF	CATGGAGCTACTCAAATGAACAAATT	Forward primer for COMT designed from accession no. HM990153
12	COMT_SyR	GACTCAACCACCCCCCAAA	Reverse primer for COMT designed from accession no. HM990153
13	CCR_SyF	GCGGCTGAGGCCAAAGT	Forward primer for CCR designed from SRA study acc. no. SRP039008 (transcript id: Locus_3266_Transcript_3/3_Confidence_0.600_Length_1300)
14	CCR_SyR	GATCCATGTAGATTGCACCGATT	Reverse primer for CCR designed from SRA study acc. no. SRP039008 (transcript id: Locus_3266_Transcript_3/3_Confidence_0.600_Length_1300)
15	CAD_SyF	AATTTGTCTCCCCAATGGTGAT	Forward primer for CAD designed from accession no. HM990152
16	CAD_SyR	GCTCTTTCATGCTTCCAATGAA	Reverse primer for CAD designed from accession no. HM990152
17	EGS_SyF	ATGGAGGAAAATGGGATGAAAAGCA	Forward primer for EGS designed from accession no. DQ372812
18	EGS_SyR	TTAAAATGCTGCTGAAGCCGGC	Reverse primer for EGS designed from accession no. DQ372812
19	EOMT_SyF	GCCTCGCGCCTCCTCTTG	Forward primer for EOMT designed from accession no. EU622048
20	EOMT_SyR	AAGTGGGATCCAAAATGACTTG	Reverse primer for EOMT designed from accession no. EU622048

Supplementary table 2. Details of primer sequences used for qRT-PCR analysis of transcription factors.

S. No.	Name of primers	Primer Sequence (5' – 3')	Details
1	bHLH_21387_RTF	AGACTCATCACCACTGCTGTTATCA	Forward primer designed from SRA study acc. no. SRP039008 (transcript id: Locus_5612_Transcript_1/6_Confidence_0.684_Length_1729)
2	bHLH_21387_RTR	CGGCACCGAGCTCATTTC	Reverse primer designed from SRA study acc. no. SRP039008 (transcript id: Locus_5612_Transcript_1/6_Confidence_0.684_Length_1729)
3	bHLH_25905_RTF	TGCCATGGAGAAACAGTCATTG	Forward primer designed from SRA study acc. no. SRP039008 (transcript id: Locus_7231_Transcript_1/1_Confidence_1.000_Length_1458)
4	bHLH_25905_RTR	CGGGACCAGAGCCATTCTT	Reverse primer designed from SRA study acc. no. SRP039008 (transcript id: Locus_7231_Transcript_1/1_Confidence_1.000_Length_1458)
5	EREB_45316_RTF	TGGCTTCTTCTGCAGCATCAT	Forward primer designed from SRA study acc. no. SRP039008 (transcript id: Locus_15957_Transcript_4/7_Confidence_0.444_Length_1969)
6	EREB_45316_RTR	AGGGAACGGCACGACGTAT	Reverse primer designed from SRA study acc. no. SRP039008 (transcript id: Locus_15957_Transcript_4/7_Confidence_0.444_Length_1969)
11	MADS-DEFB1_50254_RTF	TCCAATGGAGGACGAGCAA	Forward primer designed from SRA study acc. no. SRP039008 (transcript id: Locus_19082_Transcript_1/1_Confidence_1.000_Length_531)
12	MADS-DEFB1_50254_RTR	GCTATCCGTACAGTCGTAGAAGCA	Reverse primer designed from SRA study acc. no. SRP039008 (transcript id: Locus_19082_Transcript_1/1_Confidence_1.000_Length_531)
13	MADS-DEFB2_43518_RTF	GGCCAAAACGTAGCCAACA	Forward primer designed from SRA study acc. no. SRP039008 (transcript id: Locus_15152_Transcript_2/2_Confidence_0.800_Length_826)
14	MADS-DEFB2_43518_RTR	GGCCGCAGCTCCTCTATGT	Reverse primer designed from SRA study acc. no. SRP039008 (transcript id: Locus_15152_Transcript_2/2_Confidence_0.800_Length_826)
15	MYB2_44651_RTF	CGTCTCCGTCACTGTCAGGTT	Forward primer designed from SRA study acc. no. SRP039008 (transcript id: Locus_15644_Transcript_1/1_Confidence_1.000_Length_3464)
16	MYB2_44651_RTR	ACCTTCCGCCAGTGGTATTG	Reverse primer designed from SRA study acc. no. SRP039008 (transcript id: Locus_15644_Transcript_1/1_Confidence_1.000_Length_3464)
17	MYB3_16302_RTF	TGCATTGATTGGGCCAGTTA	Forward primer designed from SRA study acc. no. SRP039008 (transcript id: Locus_4068_Transcript_4/5_Confidence_0.733_Length_4668)
18	MYB3_16302_RTR	CGGTAAACTGATCCTCTGCAAA	Reverse primer designed from SRA study acc. no. SRP039008 (transcript id: Locus_4068_Transcript_4/5_Confidence_0.733_Length_4668)
25	MYB5_46226_RTF	TGAAAACACTGGGAATGGAGTCT	Forward primer designed from SRA study acc. no. SRP039008 (transcript id: Locus_16451_Transcript_3/3_Confidence_0.667_Length_2545)
26	MYB5_46226_RTR	CAGAGTGGTGGTTGGGAAGAA	Reverse primer designed from SRA study acc. no. SRP039008 (transcript id: Locus_16451_Transcript_3/3_Confidence_0.667_Length_2545)
27	MYC_47479_RTF	TCGGCCATGCAGTATCTTCA	Forward primer designed from SRA study acc. no. SRP039008 (transcript id: Locus_17158_Transcript_2/2_Confidence_0.667_Length_1002)
28	MYC_47479_RTR	GGCGGTAGCGAGGGAAAT	Reverse primer designed from SRA study acc. no. SRP039008 (transcript id: Locus_17158_Transcript_2/2_Confidence_0.667_Length_1002)
31	PAP1_34459_RTF	CAGCCTAATATTCCCTCCGAAAA	Forward primer designed from SRA study acc. no. SRP039008 (transcript id: Locus_10868_Transcript_1/1_Confidence_1.000_Length_1129)
32	PAP1_34459_RTR	CCGGCGTTCCCAGAGAA	Reverse primer designed from SRA study acc. no. SRP039008 (transcript id: Locus_10868_Transcript_1/1_Confidence_1.000_Length_1129)
35	TTG1_54027_RTF	GGATCATACAGCCGCTGTCA	Forward primer designed from SRA study acc. no. SRP039008 (transcript id: Locus_21367_Transcript_1/1_Confidence_1.000_Length_2013)

36	TTG1_54027_RTR	CGAGGCGAGCAGATTTCC	Reverse primer designed from SRA study acc. no. SRP039008 (transcript id: Locus_21367_Transcript_1/1_Confidence_1.000_Length_2013)
43	WRKY OB2_RTF	TGGAGTTTGCAGGCCATAGTC	Forward primer designed from accession no. DY337493
44	WRKY OB2_RTR	CCAGACCGTCCATTTCCATCT	Reverse primer designed from accession no. DY337493

Supplementary table 3. Six month essential oil profiling of *O. sanctum*, *O. gratissimum* and *O. kilimendscharicum.* Data represented as the average area percent.

	***O. sanctum* (Year- 2018)**						**P-value**	***O. gratissimum* (Year- 2018)**						**P-value**	***O. kilimendscharicum* (Year- 2018)**						**P-value**
**Compound**	**Jul**	**Aug**	**Sept**	**Oct**	**Nov**	**Dec**		**Jul**	**Aug**	**Sept**	**Oct**	**Nov**	**Dec**		**Jul**	**Aug**	**Sept**	**Oct**	**Nov**	**Dec**	
**Phenylpropenes**																					
Eugenol	62.75 ± 0.28	60.7 ± 1.41	64.68 ±0.25	62.08 ±0.59	46.54 ±1.03	38.3 ± 0.72	1.62E-10	72.± 2.03	72.7 ± 0.62	74.9± 0.38	70.3 ± 0.84	73.6± 3.97	68.4 ± 0.91	0.063	-	-	-	-	-	-	-
Methyleugenol	3.07± 0.06	0.1± 0.0	-	-	0.08± 0.01	-	1.11E-16	0.0± 0.01	0.02 ± 0.01	-	-	-	0.01 ± 0.00	3.20E-03	-	-	-	-	-	-	-
trans-Isoeugenol	-	-	-	-	-	-	-	-	0.01 ± 0.00	0.03± 0.00	0.02 ± 0.00	0.08± 0.01	-	3.63E-05	-	-	-	-	-	-	-
Estragole	0.01± 0.0	0.19 ± 0.03	-	-	-	-	2.01E-08	-	-	-	-	-	-	-	-	-	-	-	-	-	-
**Monoterpenes**																					
D-Camphor	-	0.1± 0.00	0.1± 0.00	-	0.1± 0.00	0.03 ± 0.00	4.48E-07	-	-	-	-	-	-	-	13± 3.62	56.1 ± 2.41	41.0 2± 2.10	30.0 7± 2.63	18.8 ± 2.69	28.4 ± 2.30	3.34E-06
D-Limonene	0.04± 0.00	0.08 ± 0.01	0.04± 0.01	0.02± 0.01	0.02± 0.00	0.05 ± 0.00	3.65E-05	0.0± 0.00	0.01 ± 0.00	0.01± 0.00	0.01 ± 0.00	0.01± 0.00	-	0.006	2.63 ± 0.20	4.69 ± 0.16	2.52 ± 0.25	0.95 5± 0.05	0.19 ± 0.02	0.39 ± 0.06	9.65E-09
Camphene	0.07± 0.01	-	-	0.08± 0.01	0.07± 0.01	0.08 ± 0.00	1.01E-06	-	-	-	-	-	-	-	1.51 ± 0.27	2.92 ± 0.33	1.77 ± 0.19	0.28 ± 0.08	0.17 ± 0.02	0.21 ± 0.03	1.13E-05
Endo-Borneol	0.22± 0.03	0.48 ± 0.03	0.24± 0.03	0.2± 0.01	0.18± 0.03	0.16 ± 0.01	1.57E-06	-	-	-	-	-	-	-	-	0.85 ± 0.03	0.51 ± 0.05	0.36 ± 0.03	-	-	1.06E-10
β-Myrcene	-	-	-	-	-	-	-	0.0± 0.01	0.07 ± 0.01	-	0.08 ± 0.02	0.04± 0.01	-	7.98E-06	0.32 ± 0.04	-	-	-	-	-	1.16E-08
β-Ocimene	-	-	-	0.02± 0.00	-	-	0.458	4.0± 0.09	4.05 ± 0.14	4.71± 0.09	3.77 ± 0.14	2.08± 0.60	2.42 ± 0.12	3.36E-05	0.94 ± 0.25	1.23 ± 0.32	0.55 ± 0.05	-	-	-	2.00E-04
cis-Sabinene hydrate	-	-	-	-	-	-	-	0.0± 0.00	0.34 ± 0.02	0.39± 0.04	0.64 ± 0.05	0.51± 0.03	0.31 ± 0.04	2.70E-07	-	-	0.49 ± 0.02	-	-	0.43 ± 0.01	1.14E-14
Sabinene	0.01±	-	-	-	-	-	-	-	0.18	-	0.23	-	-	0.567	-	-	-	-	-	-	-

	0.00								± 0.00		± 0.00										
α-Pinene	0.22± 0.02	0.42 ± 0.03	0.21± 0.01	0.17± 0.02	0.13± 0.01	0.32 ± 0.04	8.02E -07	0.0± 0.00	0.01 ± 0.01	0.01± 0.00	0.02 ± 0.00	-	0.03 ± 0.01	1.73E -02	0.42 ± 0.05	0.55 ± 0.04	0.39 ± 0.04	-	-	-	3.02E -08
β-Pinene	0.05± 0.01	0.09 ± 0.01	-	-	0.04± 0.00	0.05 ± 0.00	9.10E -08	-	-	-	-	-	-	-	-	0.09 ± 0.03	-	-	-	-	2.00E -04
Caryophyllene	4.31± 0.13	4.37 ± 0.16	7.38± 0.14	8.99± 0.23	8.28± 0.33	21.1 ± 0.73	3.69E -09	1.7± 0.28	1.2± 0.26	1.68± 0.14	1.12 ± 0.29	0.71± 0.10	1.71 ± 0.20	0.101	0.93 ± 0.04	1.75 ± 0.05	0.78 ± 0.07	0.58 ± 0.06	0.3± 0.08	0.9± 0.09	1.06E -05
β-Selinene	-	-	-	0.19± 0.00	-	0.14 ± 0.02	0.003	-	-	0.01± 0.00	-	-	-	0.458	-	-	-	-	-	-	-
δ-Cadinene	-	-	-	-	-	0.02	7.12E -08	0.0± 0.00	0.03 ± 0.01	0.06± 0.28	-	0.07± 0.02	0.31 ± 0.04	3.08E -01	-	-	-	-	-	-	-
Isolimonene	-	-	-	-	-	-	-	-	-	-	-	0.26± 0.00	-	0.458	-	-	-	-	-	-	-
α-Terpinolene	-	0.04 ± 0.00	-	-	-	-	0.458	-	-	-	-	-	-	-	0.53 ± 0.05	1.03 ± 0.05	0.52 ± 0.07	-	-	-	8.33E -10
β-Terpinene	-	-	0.01± 0.00	0.05± 0.00	-	-	0.401	-	-	0.07± 0.00	-	-	-	0.458	-	-	-	-	-	-	-
Phellandrene	0.01± 0.00	0.02 ± 0.00	-	-	-	0.01 ± 0.00	3.59E -06	-	-	0.18± 0.00	-	-	-	0.458	-	0.26 ± 0.03	0.23 ± 0.03	-	-	-	4.53E -09
Cymene	-	-	-	-	-	-	-	0.0± 0.00	-	-	0.06 ± 0.00	0.04± 0.01	0.02 ± 0.00	0.4	-	-	-	-	-	-	-
p-Mentha-2-en-1-ol	-	-	-	-	-	-	-	-	-	-	-	-	-	-	-	0.91 ± 0.03	-	-	-	-	5.76E -14
Neo-allo-ocimene	-	-	-	-	-	-	-	0.2± 0.03	0.21 ± 0.05	0.24± 0.05	0.25 ± 0.07	0.12± 0.02	0.15 ± 0.03	0.03	-	-	-	-	-	-	-
β- Thujene	-	-	-	-	-	-	-	0.1± 0.04	0.12 ± 0.06	0.13± 0.02	0.19 ± 0.03	0.11± 0.02	0.05 ± 0.01	0.029	-	-	-	-	-	-	-
Eucalyptol	0.06± 0.02	0.12 ± 0.02	0.09± 0.02	0.11± 0.01	0.08± 0.01	0.15 ± 0.02	0.008	-	-	-	-	-	-	-	-	-	-	-	-	-	-
ϒ-Terpinene	-	0.01 ± 0.00	-	-	-	-	0.458	0.0± 0.01	0.06 ± 0.02	0.06± 0.01	0.03 ± 0.00	0.01± 0.00	0.02 ± 0.00	8.00E -04	0.26 ± 0.01	0.48 ± 0.04	0.26 ± 0.03	-	-	-	1.36E -09
**Monoterpene alcohols**																					
4-Terpineol	-	-	-	-	-	-	-	0.0± 0.01	0.05 ± 0.01	0.04± 0.01	0.13 ± 0.02	0.09 ± 0.01	0.06 ± 0.01	0.029	0.5± 0.06	1.62 ± 0.21	0.89± 0.09	0.26 ± 0.02	0.31 ± 0.03	0.78 ± 0.04	1.46E -05
α-Terpineol	0.01± 0.00	0.02 ±	0.01± 0.00	0.01± 0.00	0.01± 0.00	0.01 ±	2.28E -02	-	-	-	-	-	-	-	-	-	-	-	-	-	-

		0.00				0.00															
β-Terpineol	-	-	-	-	-	-	-	0.3± 0.03	-	-	-	0.04 ± 0.00	-	3.98E -11	0.28 ± 0.06	0.91 ± 0.06	0.49± 0.05	-	-	-	1.20E -08
Myrtenol	-	-	-	-	-	-	-	-	-	-	-	-	-	-	0.26 ± 0.04	0.88 ± 0.06	0.41± 0.08	-	-	-	5.44E -08
Linalool	0.02± 0.00	0.14 ± 0.01	-	0.03± 0.00	-	0.03 ± 0.00	5.92E -09	0.0± 0.00	0.05 ± 0.01	0.04± 0.01	0.08 ± 0.03	0.08 ± 0.02	0.04 ± 0.01	9.70E -03	0.72 ± 0.08	1.04 ± 0.06	0.53± 0.03	-	-	0.52 ± 0.05	1.77E -08
**Sesquiterpenes**																					
epi-Bicyclosesquiphellandrene	-	-	-	-	-	-	-	-	2.36 ± 0.00	-	-	-	-	0.458	-	-	-	-	-	-	-
Humulene	0.24± 0.01	0.26 ± 0.04	0.41± 0.03	0.49± 0.03	0.45± 0.02	1.14 ± 0.07	3.24E -09	0.1± 0.03	0.09 ± 0.03	0.13± 0.03	-	0.07 ± 0.02	-	5.00E -04	-	-	-	-	-	-	-
Caryophyllene oxide	-	-	-	0.07± 0.01	0.3± 0.07	1.08 ± 0.09	3.69E -09	-	-	-	-	-	0.14 ± 0.00	0.458	-	-	-	-	-	-	-
Germacrene D	-	-	-	-	-	-	-	3.4± 0.11	0.04 ± 0.66	4.04± 0.42	2.05 ± 0.56	-	6.61 ± 0.54	1.57E -05	0.55 ± 0.06	0.89 ± 0.06	-	-	-	-	7.30E -10
β-Elemene	5.95± 0.24	5.09 ± 0.17	7.92± 0.34	9.92± 0.51	7.09± 0.51	15.7 ± 0.74	3.20E -09	0.1± 0.03	0.13 ± 0.06	0.2± 0.03	0.14 ± 0.02	0.1± 0.03	0.36 ± 0.02	0.013	-	-	-	-	-	-	-
ϒ-Selinene	-	-	-	0.25± 0.02	0.08± 0.00	-	8.91E -08	-	-	-	-	-	-	-	-	-	-	-	-	-	-
β-Bourbonene	-	-	-	-	-	-	-	0.1± 0.03	0.11 ± 0.02	0.11± 0.03	0.08 ± 0.02	0.13 ± 0.02	0.2± 0.03	0.11	-	-	-	-	-	-	-
α-Cubebene	-	-	-	-	-	0.02 ± 0.00	5.89E -06	0.2± 0.04	0.14 ± 0.02	-	-	-	-	1.62E -07	-	-	-	-	-	-	-
β-Cubebene	-	-	-	-	-	-	-	0.0± 0.02	0.06 ± 0.01	0.05± 0.01	0.12 ± 0.02	0.91 ± 0.07	0.29 ± 0.03	5.68E -10	-	-	0.32± 0.02	-	0.35 ± 0.06	-	1.07E -07
Copaene	-	-	-	-	-	0.06 ± 0.00	1.21E -12	-	-	0.22± 0.00	0.28 ± 0.04	0.23 ± 0.04	0.65 ± 0.04	2.42E -07	-	-	-	-	-	-	-
**Sesquiterpenoid alcohols**																					
δ-Cadinol	-	-	-	-	-	-	-	-	-	-	-	0.02 ± 0.00	0.15 ± 0.00	0.458	-	-	-	-	-	-	-

Data are means ± SD (at least three replicates)
